# Development of forecast models for COVID-19 hospital admissions using anonymized and aggregated mobile network data

**DOI:** 10.1038/s41598-022-22350-6

**Published:** 2022-10-22

**Authors:** Jalil Taghia, Valentin Kulyk, Selim Ickin, Mats Folkesson, Cecilia Nyström, Kristofer Ȧgren, Thomas Brezicka, Tore Vingare, Julia Karlsson, Ingrid Fritzell, Ralph Harlid, Bo Palaszewski, Magnus Kjellberg, Jörgen Gustafsson

**Affiliations:** 1 Ericsson Research, Ericsson, 164 40 Kista, Sweden; 2 Ericsson Business Area Cloud Software and Services, Ericsson, 164 40 Kista, Sweden; 3 Telia Company AB, 169 94 Solna, Sweden; 4 Department of Quality and Patient Safety, Sahlgrenska University Hospital, 413 45 Gothenburg, Sweden; 5 Department of Analysis and Project Management, Sahlgrenska University Hospital, 413 45 Gothenburg, Sweden; 6 Södra Älvsborgs Sjukhus, Hospital Management, 501 82 Borås, Sweden; 7 Department of Data Management and Analysis, Västra Götalandsregionen, 405 44 Gothenburg, Sweden; 8 AI Competence Center, Sahlgrenska University Hospital, 413 45 Gothenburg, Sweden

**Keywords:** Computer science, Scientific data, Health care, Public health, Epidemiology

## Abstract

Reliable forecast of COVID-19 hospital admissions in near-term horizons can help enable effective resource management which is vital in reducing pressure from healthcare services. The use of mobile network data has come to attention in response to COVID-19 pandemic leveraged on their ability in capturing people social behavior. Crucially, we show that there are latent features in irreversibly anonymized and aggregated mobile network data that carry useful information in relation to the spread of SARS-CoV-2 virus. We describe development of the forecast models using such features for prediction of COVID-19 hospital admissions in near-term horizons (21 days). In a case study, we verified the approach for two hospitals in Sweden, Sahlgrenska University Hospital and Södra Älvsborgs Hospital, working closely with the experts engaged in the hospital resource planning. Importantly, the results of the forecast models were used in year 2021 by logisticians at the hospitals as one of the main inputs for their decisions regarding resource management.

## Introduction

COVID-19 outbreaks have exhausted healthcare systems around the world. Concentration of admitted patients during outbreaks and limited resources at hospitals put pressure on healthcare systems. Knowing estimated number of admitted (hospitalized) patients in near-term horizons of two-to-three weeks can significantly facilitate resource management and planning. Forecasts of the number of admitted patients can serve as an important input for prediction of hospital resource allocation. However, developing forecast models for admitted COVID-19 patients has proven to be challenging^1–5^ due to, among others, lack of historical data, involvement of many external factors, and most notably the evolving nature of COVID-19 outbreaks including evolution of SARS-CoV-2^6^, dynamic nature of people behavioral response to external factors such as regulations set by authorities^7^, increasing number of people with antibodies, and evolution of antibody immunity to SARS-CoV-2^8^.

Merely considering historical data on the number of COVID-19 hospital admissions for prediction of the future number of admissions is not sufficient, and that can result in forecast models that lack the novelty factor - in the sense that they fail to capture novel trends for which there are no precedence in the past. Effectively capturing trend changes helps in proactive decision making which is vital during outbreaks. Inclusion of external factors in forecast models might improve their efficacy in capturing the novel trends. However, it is not straightforward as there are many external factors to consider and it is difficult to determine their importance^9–14^. Examples of such external factors are various temporal seasonalities (e.g., weekly and monthly seasonalities), public holidays, events, weather forecasts, regulations set by authorities, and changes in behavior of people at different phases of the pandemic. Aware of this, here, we argue in favor of using mobile network data of user activities as one of the main inputs for construction of the forecast models for COVID-19 hospital admissions in near-term horizons. We motivate use of mobile activity data for development of the forecast models by their inherent ability in capturing social behavior of people with respect to their physical movements in the society.

Inclusion of the mobile activity data, in addition to the historical data on COVID-19 hospital admissions, enables us to construct forecast models that maintain their novelty factor, leveraged on the approximate time lag between the point in time when people first come into contact with virus and the time when they are hospitalized. Our underlying hypothesis is that the user activities are positively correlated with the number of admitted patients, as the higher activity means concentration of more individuals in a limited area and in turn higher risks of exposure to SARS-CoV-2 virus.

During the COVID-19 pandemic, the use of mobile network data of user activities has seen several applications, such as to inform reopening strategies^15,16^, for informing evidence-based policy making by authorities in attempt to manage the spread of SARS-CoV-2^17–19^, early detection of COVID-19 outbreaks^20,21^, and for informing COVID-19 forecast models^22^.

In a case study, we use irreversibly anonymized and aggregated geographical grid-level hourly mobile network data of user activities in Västra Götaland county in Sweden provided by Swedish operator Telia Sverige AB, and develop forecast models for prediction of the number of admitted COVID-19 patients at Sahlgrenska University Hospital (SU) located in Gothenburg and Södra Älvsborgs Hospital (SÄS) located in Borås. We describe development of the forecast models and discuss how insights from the models were used in planning and prediction of healthcare demands and resources.

## Results

### Development of the forecast model

**Figure 1:**
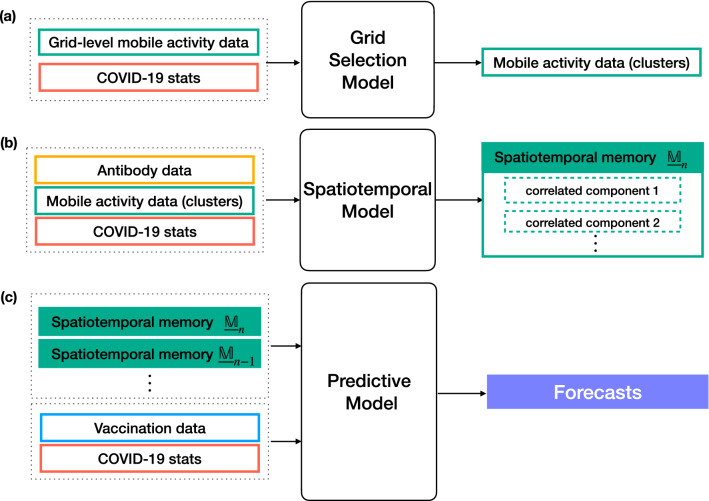
Modular description of the forecast model pipeline and its main components. (**a**) The grid selection model selects clusters of grids from mobile activity data that are best reflective of user activities in relation to COVID-19. (**b**) The spatiotemporal model uses hourly mobile activity data from selected clusters of grids and constructs a spatiotemporal memory of correlated components. These correlated components are the latent spatiotemporal patterns in mobile activity data that are either statistically positively or negatively correlated with daily number of admitted COVID-19 patients. (**c**) The predictive model uses historical frames of spatiotemporal memory matrices, together with historical COVID-19 admission data and vaccination data, in order to produce the predicted number of admitted patients for the duration of the forecast window.

Development of the forecast models for the near future prediction of the number of admitted COVID-19 patients using mobile network activity data is one of the main results of this study. Our forecast model pipeline is composed of three interconnected models, namely: the grid selection model, the spatiotemporal model, and the predictive model. Figure 1 shows the key components of the forecast model. A detailed description of the mathematical formulation and algorithmic implementations are provided in Methods.

There are three types of input data provided to the forecast model, namely: (i) historical data on the number of admitted COVID-19 patients aggregated daily per hospital, hereafter referred to as the COVID-19 admission data, (ii) external factors in the form of antibody and vaccination data, and (iii) privacy-preserving anonymized and hourly aggregated mobile activity data. The forecast model is fully data-driven which takes the three types of input data and produces prediction of the number of admitted patients for the duration of the forecast window.

We constructed two forecast models, one for SU and one for SÄS, providing daily predictions for the duration of 21 days. These two forecast models share the same underlying architecture, while being optimized separately. We proceed with briefly introducing the main three components of the forecast model pipeline.

#### Grid selection model

Mobile network data contain timeseries of aggregated hourly activities per grid in order of thousands. The grids are spread out across 49 municipalities in the Västra Götaland region, shown in Fig. 11. While hourly mobile activity data from the grids carry useful information about user activities, not all grids are equally relevant to the behavioural aspects related to COVID-19. Thus, there was a need for selection of the most relevant grids.

In construction of the grid selection model, we opted for a data-driven approach such that selected grids of interest can dynamically change over time as do user behaviors throughout the pandemic. As shown in Fig. 1a, the model takes in both historical data on grid-level hourly mobile activity data and COVID-19 admission data. It then selects clusters of grids that are most related to the user activities in connection to COVID-19.

**Figure 2:**
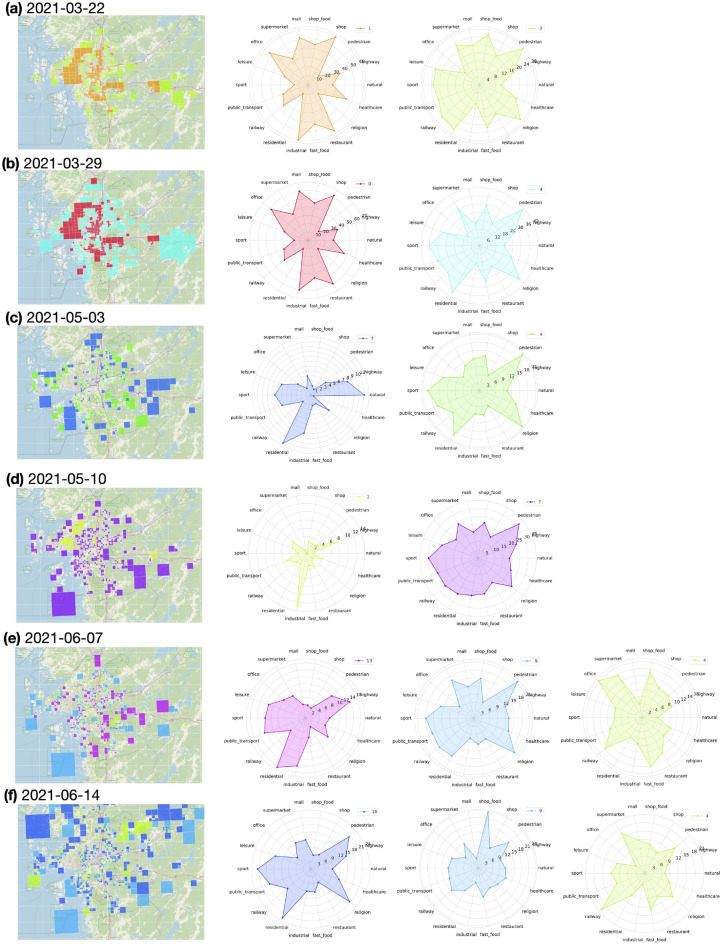
Selected clusters of grids by the grid selection model used in construction of the forecast models for Sahlgrenska University Hospital. (**a**–**f**) The figure shows the results for selected analysis dates. Note that there is no one-to-one correspondence between the same colors across two arbitrary analysis dates.

**Figure 3:**
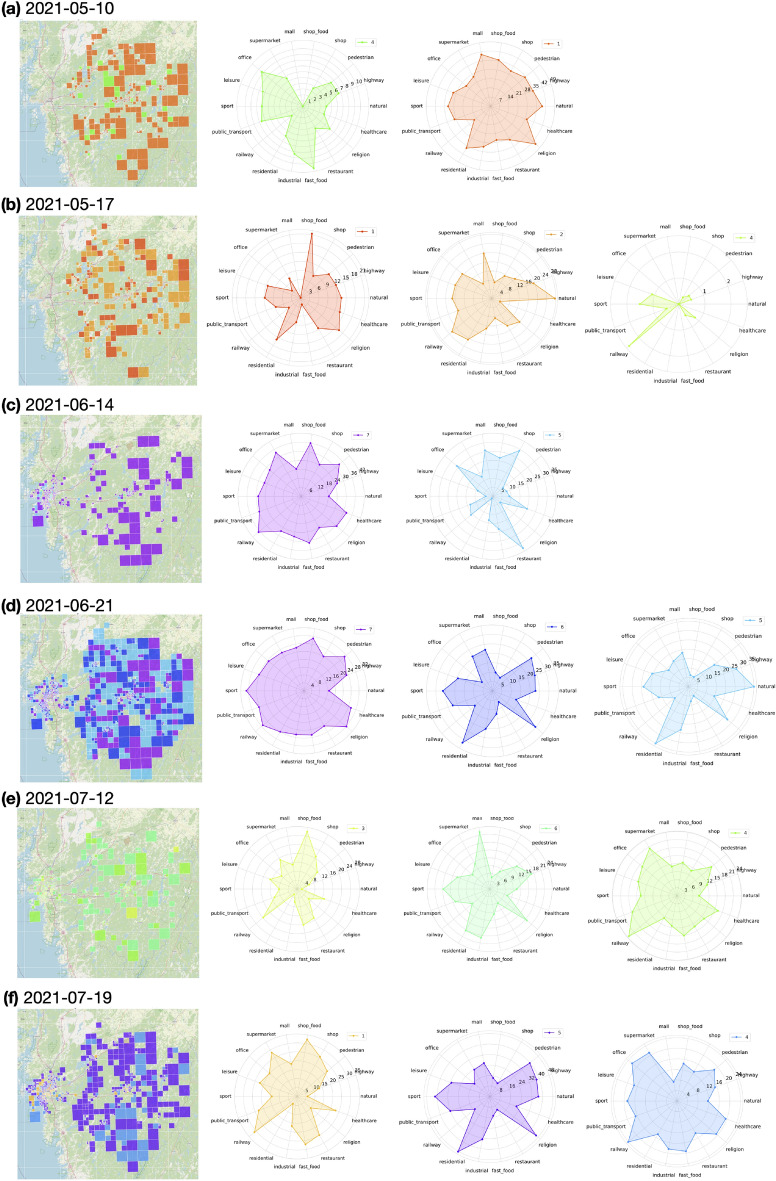
Selected clusters of grids by the grid selection model used in construction of the forecast models for Södra Älvsborgs hospital. (**a**–**f**) The figure shows the results for selected analysis dates. Note that there is no one-to-one correspondence between the same colors across two arbitrary analysis dates.

Grid selection was performed on a weekly basis as planning at the hospitals were done weekly. Figures 2 and  3 illustrate the selected clusters of grids at selected analysis dates used for construction of the forecast models for SU and SÄS, respectively. Using tags taken from OpenStreetMap^23^, one can identify the geographical objects that the selected clusters of grids represent for a given analysis date.

#### Spatiotemporal model

Hourly mobile activity data from selected clusters of grids contain useful spatial information about user activities. Additionally, these data are temporal in nature whose dynamics are affected not only by short-to-long range seasonalities, such as hourly and weekly seasonalities, but also various external factors, such as possibly evolution of antibody development and regulations set by authorities. This implies to the need for capturing temporal dynamics in modelling of such data.

Our hypothesis was that there are certain temporal patterns hidden in the mobile activity data that are particularly useful for the analysis of COVID-19. Thus, the forecast model was equipped with a spatiotemporal model. As shown in Fig. 1b, the model takes as its inputs hourly mobile activity data from selected clusters of grids, historical COVID-19 admission data and antibody data. It then constructs a spatiotemporal memory containing useful information about the short-to-long term dynamics in data. Specifically, the spatiotemporal memory contains latent spatiotemporal patterns in mobile activity data that satisfy the following two conditions: (i) they are one of the major spatiotemporal patterns in the data, (ii) and they are either statistically positively or negatively correlated with the number of admitted patients. The major spatiotemporal patterns are defined in Methods. Conceptually, the first condition ensures that only those latent spatiotemporal patterns are used for the subsequent correlation analysis that are supported by sufficient data samples.

**Figure 4:**
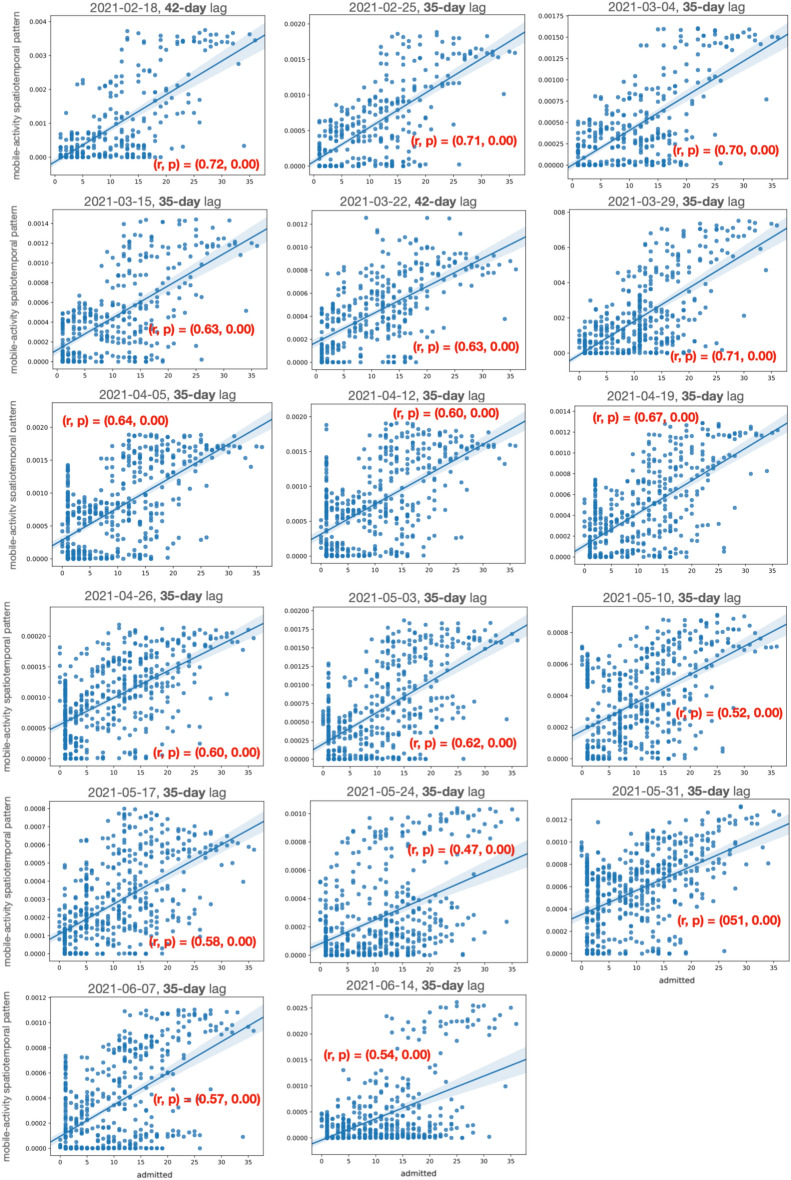
Pearson correlation between the positively correlated spatiotemporal component and the daily number of admitted patients at SU. The positively correlated spatiotemporal components are extracted from mobile activity data by the spatiotemporal model. The figure shows the correlation (r) score and the statistical significance (p) score across various analysis dates. The figures also show the optimal time lag at which the Pearson correlation between the spatiotemporal components and daily number of admitted COVID-19 patients is at its highest value. The optimal time lag is 35 days for all analysis dates except for 2021-02-18 and 2021-03-22 which is 42 days.

**Figure 5:**
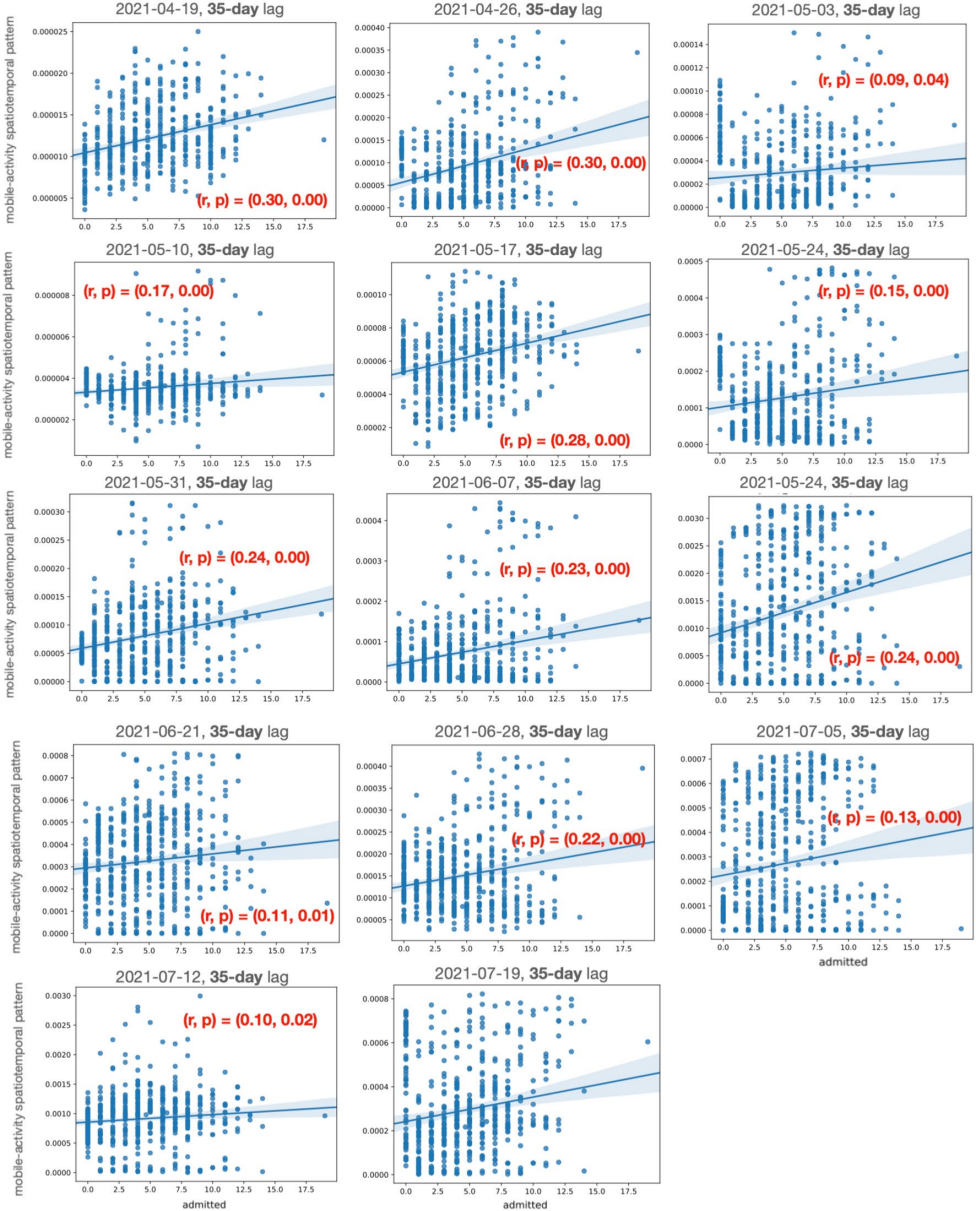
Pearson correlation between the positively correlated spatiotemporal component and the daily number of admitted patients at SÄS. The positively correlated spatiotemporal components are extracted from mobile activity data by the spatiotemporal model. The figures show the correlation (r) score and the statistical significance (p) score across various analysis dates. The figures also show the optimal time lag at which the Pearson correlation between the spatiotemporal components and daily number of admitted COVID-19 patients is at its highest value. The optimal time lag is 35 days for all analysis dates.

Figures 4 and  5 show the (Pearson) correlation between the positively correlated spatiotemporal patterns and the daily number of admitted patients at SU and SÄS, respectively.

#### Predictive model

The forecast model is equipped with a predictive model in the form of a regressor. As shown in Fig. 1c, the predictive model takes as its inputs all available historical frames of the spatiotemporal memories, historical data on vaccination data, and historical COVID-19 admission data. It then produces predictions for the number of admitted patients for the duration of the forecast window, 21 days.

### Considerations in development of the forecast model

#### Validation of the forecast models

Validation of the forecast models for COVID-19 was challenging due to the dynamic nature of pandemic. We took the following approach for the validation of the forecast models. For a given analysis date, we divided available historical data into a train set and a validation set. The forecast model parameters were tuned guided by the results on the validation set. We varied the size of the validation set, from one week to six weeks to find the best setting for the parameters of the forecast model. The setting of the parameters that performed well on average across all validation sets were used for the final analysis, referred to as the optimal parameter setting. Next, we trained the model on the entire historical data, using the optimal parameter setting, which provided final forecasts for the duration of the forecast window. Such validation procedure was performed on a weekly basis for both SU and SÄS forecast models.

#### Evaluation of the forecast models

Evaluation of the forecast models was done based on both visual inspection by healthcare subject matter experts and objective measures. The visual inspection of the forecasts was done to examine model performance in capturing important trends in data. We found that using primarily objective measures for the evaluation of the forecast models while useful can be sometimes misleading. As an example, a forecast model can miss out on capturing important trend changes while yet achieving reasonable performance based on the objective measures. It was found that the visual inspection of the predictions for the evaluation of the forecast models, by healthcare experts, can provide complementary insights.

#### Addressing the degeneracy problem of the forecast models

Training the forecast models involved minimizing a loss function between true and predicted number of admitted patients. We found that the forecast models often fall into degenerate solutions. The problem of degeneracy of a forecast model arises when a forecast model learns to “repeat the past” and by doing so it achieves misleadingly a low loss. This may be explained by noting that the COVID-19 admission data can be seen mostly as stationary signals containing relatively long and steady-state regions followed by sudden rare increasing or decreasing trends. The main issue with a degenerate model is that it is inherently unable to predict novel trend changes resulting in unreliable forecasts.

To reduce the degeneracy problem, we introduced a regularization to the loss function of the forecast models. The regularization was designed to discourage forecasts that are similar to the past and encourage uncovering novel trends. The addition of the regularization was the key in reducing the degeneracy problem in our forecast models for SU and SÄS. Construction of the regularization is discussed in Methods.

#### Inclusion of the external factors in the forecast model

As stated earlier, our main hypothesis in using mobile network data is that user activities are positively correlated with the spread of SARS-CoV-2 virus. However, as the antibody rate increases in population, user activities captured by the mobile activity data become less correlated with the number of admitted patients. The basis for this assumption is that the majority of individuals with antibodies would likely develop light symptoms which would not lead to hospitalization. To compensate for the effect of the antibody development in reducing predictive capabilities of the mobile activity data in relation to COVID-19, we considered antibody test data and vaccination data as the two external factors. However, between the two, vaccination data were given higher importance by the forecast model. For the case of the antibody test data, they were included indirectly through the spatiotemporal model while for the case of the vaccination data, they were included directly through the predictive model, as shown in Fig. 1. In Methods, we describe the exact mathematical formulation used for including the external factors in the forecast models.

### Forecasts of the number of admitted patients at SU and SÄS

**Figure 6:**
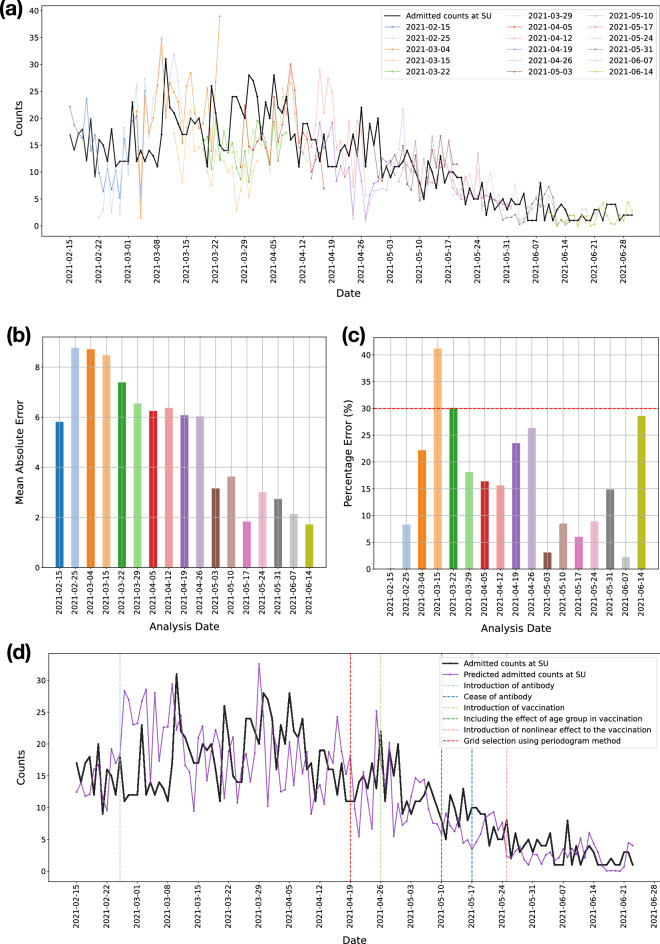
Prediction of the number of admitted COVID-19 patients at Sahlgrenska University Hospital (SU). Forecast models were run regularly for 17 analysis dates (deliverable dates) starting from February 15, 2021 until June 14, 2021. (**a**) Forecasts from the 21-day forecast model at each analysis date. (**b**) Error in prediction in terms of the mean-absolute-error between true and predicted number of admitted patients, averaged across the duration of the forecast window. (**c**) Error in prediction in terms of the percentage error score per analysis date, averaged across the duration of the forecast window. Across all analysis dates, the total number of admitted patients were more than 15 patients. In 16 out of 17 analysis dates, the error is less than 30%. (**d**) Prediction of the number of admitted patients considering forecasts from latest models, built using the latest available data. The evolution of the forecast models is highlighted with markers indicating the major changes to the forecast model.

Figure 6a shows the predicted number of admitted patients at SU during course of pandemic from February 15, 2021, until June 23, 2021, provided by the 21-day forecast model. Forecasts were delivered as inputs to SU on a weekly basis in 17 analysis dates (deliverable dates). That means the forecast models were run at various analysis dates while providing predictions for the duration of the next 21 days. Figure 6b,c show the error in prediction per analysis date in terms of the mean-absolute-error (MAE) score and the percentage error (relative error) between true and predicted number of admitted patients, averaged across the duration of the forecast window. For the purpose of hospital resource planning, the forecasts from the most recent models were used by logisticians at SU. The most recent model is referred to the model built on using the latest available data at the time. Figure 6d shows forecasts from the latest models. The evolution of the forecast models is highlighted with markers indicating the major changes to the forecast model.

**Figure 7:**
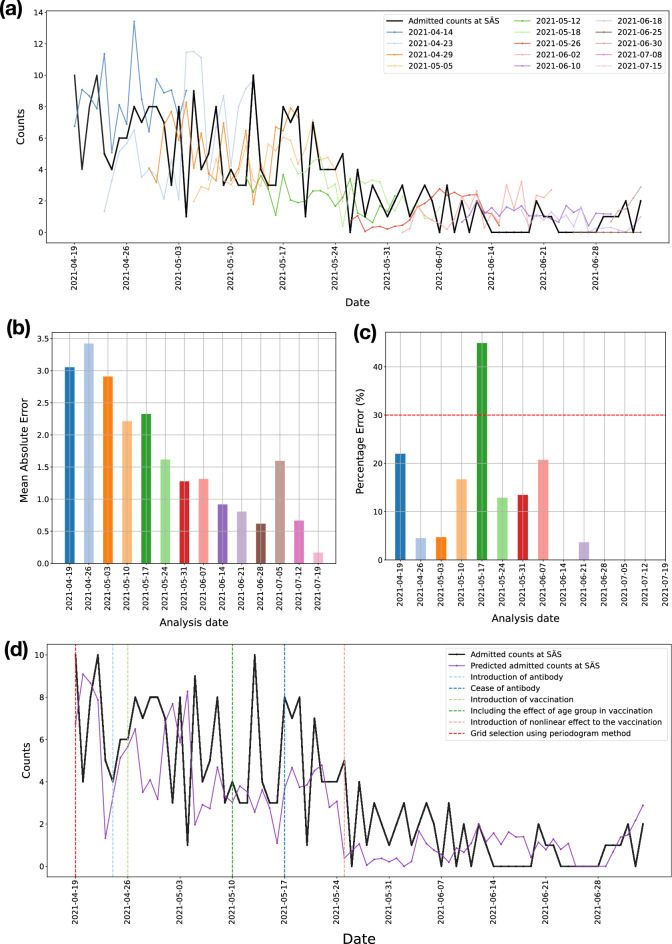
Prediction of the number of admitted COVID-19 patients at Södra Älvsborgs Hospital (SÄS). Forecast models were run regularly for 14 analysis dates (deliverable dates) starting from April 19, 2021 until July 19, 2021. (**a**) Forecasts from the 21-day forecast model at each analysis date. (**b**) Error in prediction in terms of the mean-absolute-error between true and predicted number of admitted patients, averaged across the duration of the forecast window. (**c**) Error in prediction in terms of the percentage error score per analysis date, averaged across the duration of the forecast window. Percentage errors are not shown for those analysis dates for which the total number of admitted patients were fewer than 15 patients. In 8 out of 9 analysis dates, for which the total number of admitted patients were more than 15 patients, the error is less than 30%. (**d**) Prediction of the number of admitted patients considering forecasts from latest models, built using the latest available data. The evolution of the forecast models is highlighted with markers indicating the major changes to the forecast model.

Similarly, Fig. 7a shows the predicted number of admitted patients at SÄS from April 19, 2021, until July 4, 2021, provided by the 21-day forecast model. Forecasts were delivered as inputs to SÄS on a weekly basis in 14 analysis dates. Figure 7b,c objectively evaluate the error in prediction per analysis date in terms of the MAE score and the percentage error between true and predicted number of admitted patients, averaged across the duration of the forecast window. Figure 7d shows forecasts from the latest models provided by the 21-day forecast model which were used by logisticians at SÄS for the resource-planning purpose.

**Figure 8:**
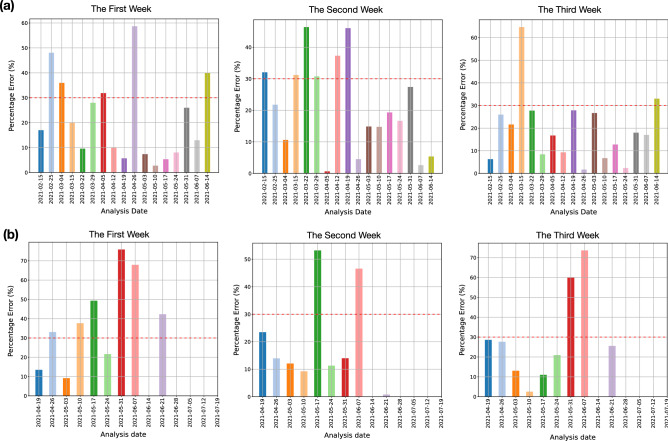
Error in prediction in terms of the percentage error score per analysis date. The duration of the forecast window of 21 days is partitioned into three parts, namely, the first week, the second week, and the third week. (**a**) Percentage error per partition and per analysis date, averaged across 7 partition days, for SU and (**b**) for SÄS. Percentage errors are not shown for those analysis dates for which the total number of admitted patients were fewer than 15 patients.

Alternatively, we can study the quality of the forecasts by partitioning the forecast window into three separate weeks. Figure 8 shows the averaged percentage error per partition for SU and SÄS.

## Discussion

The goal of the project was to provide insights that help hospitals in resource management and planning during the COVID-19 pandemic. We hypothesized that privacy-preserving mobile network data of user activities, that are irreversibly anonymized and aggregated, were reflective of the social activity of people in terms of their physical movement in the society. To validate our underlying hypothesis, we took a model-based approach as we believed the aspects of the mobile network data that are of interest for the analysis of COVID-19 are latent in the data. Thus, the idea was to extract those latent spatiotemporal patterns in mobile activity data that are of utmost relevance to the analysis of COVID-19 admission data.

The first step in achieving this goal was to extract the spatial information by selecting the geographical grids of interest to COVID-19. We first considered possibility of a hypothesis-driven approach in selection of the grids based on their geographical locations. However, the hypothesis-driven approach would not have taken into account the dynamic nature of people behavioural response to the evolving pandemic situation. As an example, restrictions set by authorities affect people social behavior and that in turn affects activities registered by the grids, differently. Therefore, instead of a hypothesis-driven approach, we decided to opt for a data-driven approach in selection of the grids. Figures 2 and  3 show that the selected clusters of grids - and what they represent - change dynamically throughout the pandemic. As an example, the effect of season change in people behavior has been captured in the selected grid clusters such that in winter time grids of interest are mostly concentrated around city center areas while in summer times, in addition to the city center areas, there are clusters of grids representing areas outside of the city such as parks and cottage areas.

The spatially relevant grid clusters were in the form of timeseries and it was important to capture as well those temporal dynamics in the data that are relevant for the analysis of COVID-19 admission data. We modeled the timeseries using the spatiotemporal model by decomposing data into a number of spatiotemporal patterns representing various temporal dynamics in the timeseries data. We then showed that there are indeed latent spatiotemporal patterns in mobile activity data that are statistically correlated with the number of admitted patients at the hospitals. Figures 4 and 5 show the correlation scores for the positively correlated spatiotemporal patterns throughout the pandemic for SU and SÄS, respectively. We observed that the correlation scores were considerably higher for SU than SÄS. This could be explained partly by the fact that SU is a larger hospital and its catchment area includes municipalities with higher population densities than the ones for SÄS. Hence, user activities captured in mobile activity data are better reflective of the people behavior.

In spatiotemporal modelling of the mobile activity data for the extraction of the correlated spatiotemporal patterns, we considered various lags between mobile activity data and the number of COVID-19 admitted patients. The lag duration was varied from 7 to 49 days with a step size of 7 days. For different lags, we computed Pearson correlation between spatiotemporal patterns extracted from mobile activity data and the number of admitted patients. We found that higher correlation scores were achieved for longer lags between 28 days and 45 days while, in most cases throughout the pandemic, the highest correlation score was achieved for the 35-day lag. This is shown for SU in Fig. 4 and for SÄS in Fig. 5.

Leveraged on the predictive capabilities of the correlated spatiotemporal patterns, we built the predictive model of the number of admitted patients which uses these patterns as one of the main input features in addition to the historical COVID-19 admission data. We found the spatiotemporal patterns having a complementary role which proved useful for construction of our 21-day forecast models. This is explained as follows. Historical data on the number of admitted COVID-19 patients are better predictive of the future number of admitted patients for shorter lags (lags smaller than 7 days), and they lose their predictive relevance as the lag increases beyond 14 days. However, mobile activity data were shown to be most relevant for longer lags (28 to 42 days) but to have relatively limited predictive relevance for shorter lags, smaller than 7 days. Taking into account these two input features concurrently helped the forecast models to harvest useful information for near-term (i.e., 21 days) prediction of the number of admitted COVID-19 patients.

In addition to the historical COVID-19 admission data and mobile activity data, the forecast models were enriched with additional inputs provided by the external factors related to the development of the antibody in population, namely antibody test and vaccination data, when they became available. Purposefully, we did not include effect of the external factors that are implicitly captured in mobile activity data such as weather condition, season, public transportation, and compliance to the regulations set by authorities. As an example, in the latter case, the implicit assumption is that the mobile network data of user activities is a proxy of compliance to the regulations.

It is important to note that the forecast model pipeline was developed during the pandemic. As the pandemic evolved, we needed to make changes to the forecast model. This is referred to as the evolution of the forecast model pipeline. Major changes to the models are highlighted in Figs. 6d and 7d. The changes to the forecast models are mostly related to the introduction of external factors to the forecast models. Antibody test data were included in the forecast models for SU on 2021-02-25 and for SÄS on 2021-04-24. However, later on, the free-of-charge offering of the test for inhabitants was discontinued. The associated cost with the test could have imposed biases in our forecast models, as the statistics on the antibody rates may not have been representative of the whole population. Therefore, the decision was made to not use these data for the subsequent analyses, effectively from 2021-05-17 onward for both SU and SÄS forecast models. Vaccination data were included in the forecast models of SU and SÄS on 2021-04-26. Initially, we did not have access to the age groups. From 2021-05-10, the effect of age group of the vaccinated population was included in the models as such data became available to us. Prior to 2021-05-25, we used linear effect in inclusion of the vaccination data. Since 2021-05-25, we used nonlinear effect where the non-linearity was learned from vaccination experience in Israel^24^, as described in Methods. In terms of the methodology, the only major change to the forecast model pipeline was related to the grid selection model. Since 2021-04-19, we changed the method of grid selection from the distance correlation to the periodograms, as described in Methods. The transformation of the timeseries data to periodograms added reliability and freedom to choose the seasonality related frequencies in the data reflecting the latest state of the pandemic.

Forecast models for SU and SÄS were run regularly on a weekly basis as deliverables to the hospitals. At each deliverable, the forecasts for the duration of 21 days were provided to the logisticians at the respective hospitals. Figures 6 and 7 summarize the results. Crucially, in 16 out of 17 deliverables to SU, the percentage error, averaged across the duration of the forecast window of 21 days, was below 30% (Fig. 6c). In the case of SÄS, excluding the analysis dates where the total number of admitted patients were fewer than 15 patients, in 8 out of 9 deliverables, the percentage error was less than 30% (Fig. 7c).

In development of the forecast models, we have made several assumptions with respect to the input data, namely, data from external factors (i.e., antibody test and vaccination data), COVID-19 admission data, and mobile network data. As discussed earlier, through evolution of the forecast models, some of these assumptions were addressed - as an example effect of antibody development in the population was included in the forecast models through inclusion of vaccination data when they became available. However, some other assumptions remained throughout, which we believe addressing them could have improved the quality of the predictions from the forecast models. In the following, we state a few important examples of such assumptions. The first category of assumptions is with respect to the mobile network data of user activities. In this study, we used data provided by Swedish operator Telia Sverige AB. Telia has the largest market share based on the number of mobile subscriptions in Sweden with about 34.6 percentage. We have made an assumption that the data from Telia are representative of the population. However, this assumption may have introduced potential bias in our analysis specially considering the age group of the base subscribers. Furthermore, the mobile network data does not include activity of the users that are solely connected to Wi-Fi. Finally, there are other sources of uncertainty in mobile network data stem from missing values, privacy-preserving aggregation procedures, possible noise in data collection, and social or regulatory recommendations that advise users to refrain from using their phones. Aware of such limitations and inherent uncertainties in mobile network data, we showed that such data can provide useful insights about the global trends of user activities. The next assumption was with respect to the data from PCR testing which was not used by the forecast models. We believe including such data in the models, as an additional input, could have helped the forecast models - particularly, if such data were available at an early stage and were made on a population basis on all individuals presented with symptoms of COVID-19. In Västra Götaland county in Sweden, different vaccines were used including Comirnaty from Pfizer, Spikevax from Moderna and Vaxzevria from AstraZeneca. However, we were not provided with the exact information about the vaccine types. Thus, in using vaccination data in the forecast models, we assumed the same efficacy for all types. Information about the mutations and variants of SARS-CoV-2 virus, and the impact of evolving mutations and variants on COVID-19 vaccines were not considered in the models. Finally, in addition to mobile network data, including data from other sources such as wastewater could have potentially enriched the models. Authorities involved in the hospital resource management and planning were regularly informed about the assumptions made in the development of the forecast models and their limitations.

At SU, forecasts of the number of admitted COVID-19 patients were primarily processed by logisticians, as one of the key inputs for prediction of hospital beds for patients with COVID-19. It was done heuristically by adding the average of days the COVID-19 patients are hospitalized to each hospitalization case provided by the forecast model. Logisticians then could calculate the number of beds needed to take care of these patients. In addition to the forecast of admitted COVID-19 patients, at SU other inputs were used for the hospital resource management. These inputs varied over time because of changes in behavior of the population. The inputs used at SU for the longest of time was analyzing the increase and decrease of positive PCR-tests from primary care (Vårdcentral) in Gothenburg which is the area where the patients hospitalized at SU live. Other inputs used during the pandemic were for example increase or decrease in number of travelers at Västtrafik, which is the public transport company in Gothenburg and calls to Vårdguiden 1177 with the symptoms that are correlated to COVID-19 (Vårdguiden 1177 is a Swedish service providing healthcare by telephone and the central national infrastructure for Swedish healthcare online). SU also followed the content of virus in sewage water as yet another factor in their considerations^25,26^. These different inputs were weighted together and then presented to the group in charge of the hospital resource management. This group subsequently made decisions whether to open or close wards and beds dedicated for patients with COVID-19. At SÄS, the forecasts on the number of admitted patients were used together with other indicators such as number of positive PCR in the community and cluster outbreaks in part of the region in order to make an estimation whether the number of admitted patients would increase, decrease or remain stable for the next 14-21 days. This estimation was used to adjust the estimated number of beds available for COVID-19 patients.

Collaboration between operative and academic departments have proved to be a key factor of success in the presented study. At the hospital level, there was a profound knowledge of how the disease itself influenced the need of both intensive care resources as well as of ordinary care facilities. It was observed that a rather constant factor of the admitted patients who were hospitalized needed intensive care (approximately 15%) and a higher fraction of the beds were occupied during the high waves of the pandemic by the same patients (approximately 25%). This insight called for a need of being able to estimate the number of patients that were admitted from time to time in order to always being able to correctly allocate resources to all patients who were imperatively in need of hospital care. SU found it extremely important to be able to continuously (on a weekly basis) forecast the number of admitted patients.

Collaboration with the academics and industry was regarded as a necessity in providing new opportunities for developing models that later proved very useful. The collaboration was performed with an open mind for the skill in each area that the different actors could provide. This resulted in a dynamic evolution of the knowledge of how models that could be of use could be produced. There were no economic or other constraints, such as a pre-designed overall protocol of research in the collaboration which allowed for free thinking that we feel is of great importance for developing this kind of models. Due to the immense complexity of a pandemic, such constraints would rather hinder than facilitate research that had to be performed at a reasonable pace in order to be operationally useful as the pandemic developed in its own unpredictable way. From the hospital perspective we have learned a lot from both the way the collaboration was initiated and performed and what kind of data we think would be of great use in future pandemic situation, in order to forecast the need of hospital resources. Decision makers can draw important insights from this work in early-stage formulation of sustainable strategies when it comes to recommendations to inhabitants how to behave, how to proceed with shut downs of municipal services, and providing healthcare region testing for infection at a high level.

This project has been a collaborative effort between the two hospitals (SU and SÄS) and the private companies (Ericsson and Telia). It was initiated as an effort to handle the difficult and alarming pandemic situation. The rapid project initiation and the positive project outcome show the importance of forming and maintaining active networks across industries, both private and public sectors. The project can be seen as an excellent example of how society can benefit from digitalization, for example, mobile phones, mobile networks and data-driven model development. An interesting aspect of the project was that the project outcomes could be used timely in operational plannings. This was at first through insights from visualization of the mobile network data. However, as the project progressed, more advanced outcomes (the forecast models) were generated and used gradually in practice. Throughout the project, close communication between the parties was prioritized and maintained at various stages of the project including problem formulation, interpretation of the results, limitations and proper usage of the forecast models.

Finally, the results would not have been possible without the close three-party collaboration, and certainly not in such a timely fashion that it was used while the pandemic was still ongoing.

## Methods

### Mobile activity data

Mobile activity is based on the signaling data between mobile phones and mobile cell towers. Data used in this study were provided by Swedish operator Telia Sverige AB containing hourly mobile activities in 49 municipalities located in Vvstra Götaland county in Sweden. Hourly activities are obtained from user equipments (UEs) and are aggregated at the grid level. A grid is defined as a geographical square-like area. The grid size (spatial resolution) is determined by the location of cell towers combined with the density of cell towers and the number of signals from mobile phones. The highest spatial resolution is $500\times 500$ (m) and the lowest resolution is $16\times 16$ (km). Figure 11 visualizes 3720 grids in 49 municipalities located in Västra Götaland county in Sweden. In this study, we concentrated on two parts of the Västra Götaland county namely Gothenburg (with a population of around 625000 inhabitants) and Borås (with a population of around 117000 inhabitants), where Sahlgrenska University Hospital and Södra Älvsborgs Hospital are located, respectively. Specifically, we limited the studied areas to cities including inner city, suburbs, rural-urban and frequently visited areas by inhabitants detected from the Swedish operator Telia’s travel matrix data^17^.

The raw data were made privacy-preserving through a procedure consisting of anonymization and aggregation, extrapolation, and spatial-temporal aggregation^17^. The anonymization uses mechanism, among other mechanisms, of *k*-anonymity of 5, that is, at all steps during the process there must be at least five mobile devices or otherwise the information is discarded automatically. Aggregation follows and then numbers are further adjusted to be representative for the full population. These estimated total number of user activities are obtained per grid on an hourly basis. An activity in this context is defined as a unique dwell within a grid of at least 20 minutes^17^.

**Figure 9:**
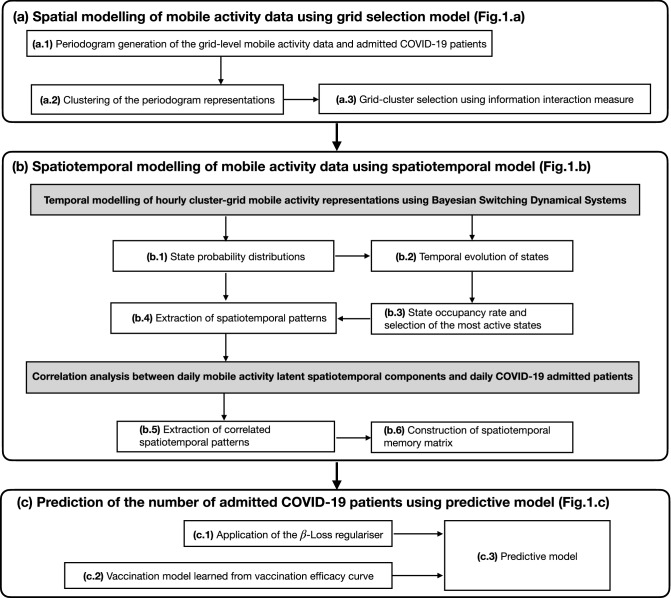
Overview of the main components in the forecast model pipeline and their functionalities.

**Figure 10:**
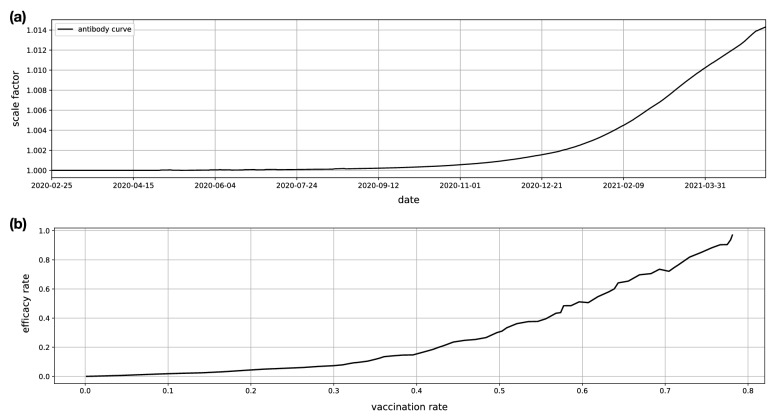
External factors. (**a**) Antibody curve which is computed from cumulative statistics on the antibody positive rates. (**b**) Vaccination efficacy curve obtained from Israel vaccination campaign data available at the time^24^.

**Figure 11:**
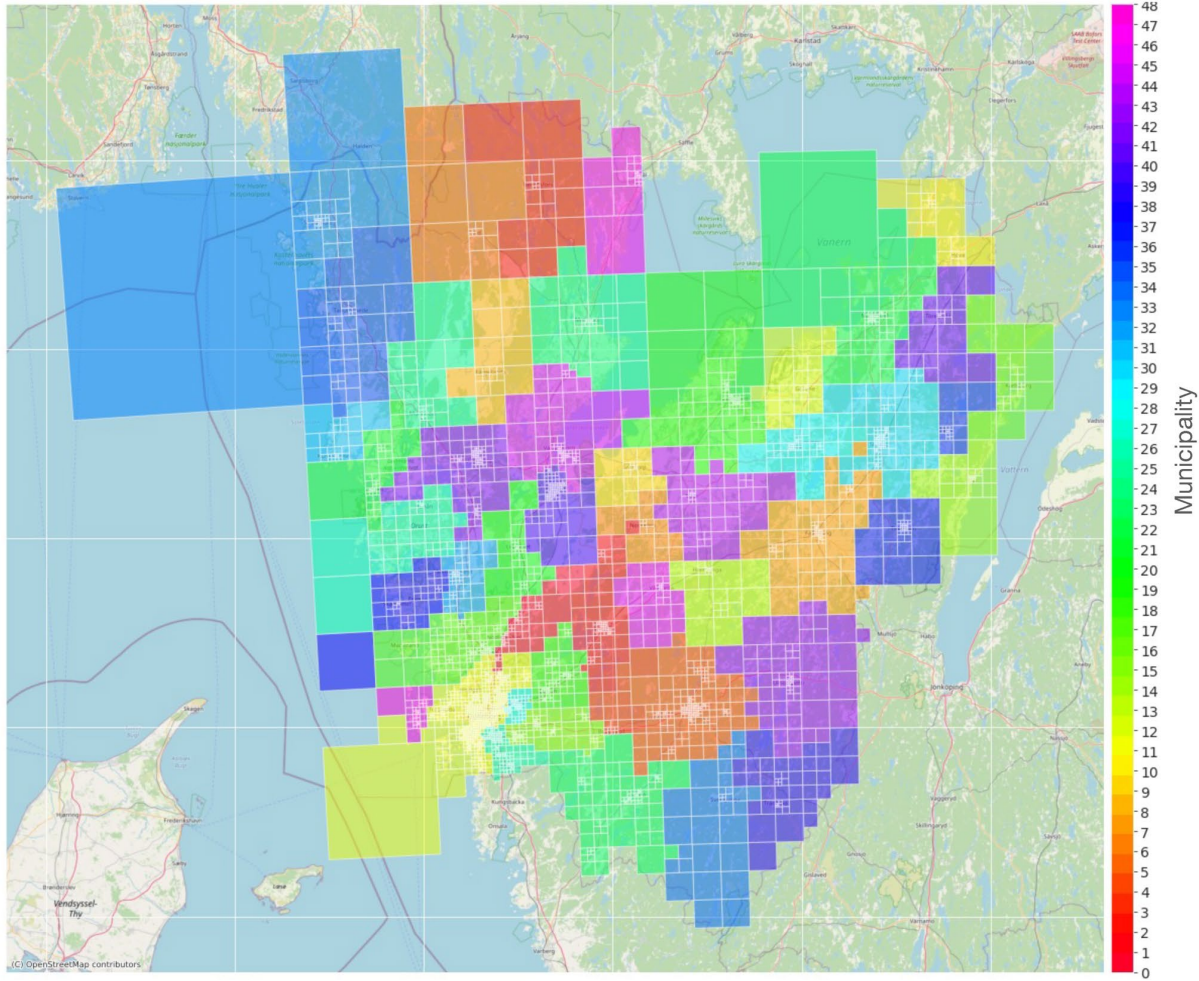
The visualization shows 3720 grids belonging to 49 municipalities located in Västra Götaland county in Sweden provided by Swedish operator Telia Sverige AB.

### Vaccination data

Aggregated vaccination data were supplied by Region Västra Götaland. In all analysis, we considered only the effect of dose one.

### Antibody data

From October 2020, inhabitants in Region Västra Götaland could receive an antibody test to verify whether they have antibodies for COVID-19. While initially, the test was free of charge, in April 2021, the free-of-charge offering of the antibody test was discontinued. The antibody test data were used by the forecast models only when they were free of charge.

### COVID-19 admission data on the number of admitted patients

At SU, for a patient to be counted as an admitted COVID-19 patient, there should be a positive PCR-test at the earliest of 14 days before the hospitalization. Therefor the number of admitted COVID-19 patients consists of not only patients confirmed with COVID-19 at the hospital when they seek care but also patients who have been tested positive for COVID-19 at for example a primary care center before seeking care at SU.

At SÄS, data on the daily number of COVID-19 admitted patient were provided by SÄS patient registering system. Patients that came to the emergency ward and were categorized as “pandemic cause” were tested and if found positive they were registered as admitted for COVID-19. The data are not public.

### Preprocessing of mobile activity data

The mobile activity data were collected from geographical grids with dynamic sizes^17^. It was decided to normalize the data by the respective grid area. This changed the data units from raw mobile activity counts to mobile activity per square meter.

The outlier removal was a part of the cleaning procedure and was performed on the raw mobile activity data. It was based on two statistical concepts: (i) the median absolute deviations (MAD)^27^, and (ii) kurtosis score computed from the historical data. A data point was marked as an outlier when its MAD distance and kurtosis score were greater than the respective thresholds.

Due to existence of trend changes (or concept drifts) in mobile activity data, we used double median absolute deviation (left-and-right MADs). The left-MAD was used to calculate the distance from the median of all points less than or equal to the median while the right-MAD was used to calculate the distance for points that were greater than the median. Thus, the MAD threshold was calculated as:

1 $$\begin{aligned} \Delta = \frac{\Lambda ({X})}{\Gamma (X)}, \end{aligned}$$

where $\Lambda ({X})$ is absolute deviation of the timeseries *X* and $\Gamma (X)$ is the double MAD of *X*.

For a given data point, a kurtosis score was calculated using the historical data including the current data point. If the kurtosis score was larger than the threshold, the data point was flagged as the outlier. The threshold for the kurtosis was set experimentally to 3 which corresponds to the kurtosis value for a univariate normal distribution.

No handling of the missing data were required during preprocessing step of the mobile activity data due to the fact that our grid selection model could handle missing data.

### Grid selection model

As shown in Fig. 1a, the grid selection model takes as its inputs (i) hourly mobile activity data from all grids, and (ii) daily COVID-19 statistics on the number of admitted patients. It then provides the hourly mobile activity data from grid clusters that are best reflective of user activities in connection to COVID-19 admission data. The main components of the grid selection model are shown in Fig. 9a. Here, we describe these components.

At the first step, we convert both mobile activity timeseries and COVID-19 admission data into periodograms using Lomb-Scargle approach^28^. The mobile activity data contained missing values due to, among others, global mobile network outage, problems with data collection, and mobile network maintenance. The duration of missing values varied from one to several days. The use of Lomb-Scargle approach was motivated by the fact that it can effectively handle the missing data. In one study^29^, it is shown to have reasonably high tolerance for the presence of the missing data up to approximately 30%. However, except in rare occasions, we had far fewer missing data. In cases where missing data were simply too many to handle by Lomb-Scargle approach, the affected grids were discarded. For our analysis, we decided to use the log-periodogram representations which are computed by taking natural logarithm of the periodograms. The use of logarithmic representations helps reduce the high-frequency noise in periodograms due to the measurement noise. To further reduce high-frequency noise in log-periodograms, a Butterworth low pass filter was applied to the representations.

Next, the log-periodogram representations were clustered using Ward hierarchical clustering approach^30^. To find the optimal number of clusters, we used the information theoretic measure of interaction information^31^, which is a multivariate generalization of the mutual information. For this purpose, the interaction information was calculated between the cluster centers and COVID-19 admission data used as the prediction targets. The negative interaction was chosen as the criterion for selection of the number of clusters. The selection of the negative interaction over the positive interaction was experimentally verified. The number of clusters which resulted in the highest negative interaction information score was selected as the optimal number of clusters. Supplementary Fig. 1 exemplifies the grid clusters resulting from the clustering step. Not all these clusters are reflective of people behavior in connection to COVID-19. The final step was to select clusters of interest defined as grid clusters that are best related to COVID-19 admission data. We selected the smallest set of clusters with the highest negative interaction information.

#### Evolution of the grid selection model

The method of grid selection changed over the course of pandemic, as highlighted in Figs. 6d and  7d. The changes to the grid selection model are related to use of the timeseries conversion of the grids to the periodograms from 2021-04-19. Initially, the Pearson correlation distance was used to cluster the timeseries. However, the distance metric based on the Pearson correlation showed to be unreliable in combination with constantly growing size of the timeseries due to addition of new data on a weekly basis. The transformation of the timeseries data to periodograms added reliability and freedom to choose the seasonality related frequencies for clustering. Additionally, it helped to test the timeseries clustering with different duration and allowed to find the best time duration reflecting the latest state of the pandemic. Thus, we selected several data chunks with different time duration, from 4 to 24 weeks into the past. These data chunks were clustered separately. The result was several clustering configurations. The clustering with the highest negative interaction information including the COVID-19 admission data was used for the subsequent selection of the cluster combination.

### Spatiotemporal model

As shown in Fig. 1b, the spatiotemporal model takes as inputs (i) the hourly mobile activity data from selected grid clusters, (ii) antibody test data, and (iii) daily COVID-19 statistics on the number of admitted patients. It then constructs a memory matrix of the spatiotemporal patterns in mobile activity data that are best related to the number of admitted COVID-19 patients, where the degree of relevance is measured in terms of the Pearson correlation score between the two. The main components of the spatiotemporal model are shown in Fig. 9b. Here, we describe these components and visualize them through an example - the example taken here is for the analysis date on 2021-06-07 for SU, and similar procedures were taken for other analysis dates for SÄS and SU.

At the first step, we obtain cluster representations of the grid clusters, where each cluster representation is the cluster’s global center obtained by taking average of the grid timeseries in the cluster. The cluster representations are hourly mobile activity data averaged across grids, referred to as the hourly cluster-grid mobile activity representations. As an example, the grid clusters and the hourly cluster-grid mobile activity representations are shown in Supplementary Fig. 2.

Next, these representations are modeled using Bayesian switching state space (BSDS) model^32^. BSDS is a temporal model which models data through a number of states. The states are modeled by Gaussian distributions where each state is fully described by the mean and covariance matrix of its corresponding Gaussian distribution. States are connected to each other through the first-order Markov chain, modeled via hidden Markov model (HMM). Throughout the analysis, maximum number of states was set to 10 where we then relied on the Bayesian model selection in BSDS for deciding on the optimal number of states. Specifically, in Bayesian model selection of BSDS, those states that have insignificant contributions in describing data are assigned weights approaching zero and are pruned out by the model automatically.

Measures extracted from BSDS that we use here include occupancy rate, temporal evolution of states, and state posterior probabilities. For our working example, Supplementary Fig. 3a shows the state distributions where each (remaining) state is presented by a Gaussian distribution. Supplementary Fig. 3b shows the state posterior probabilities from which the temporal evolution of states are obtained, shown in Supplementary Fig. 3c. The temporal evolution of states indicates which state is active at a given time and at a given day. Supplementary Fig. 3d shows the occupancy rate of each state, computed from temporal evolution of states. The occupancy rate of states indicates the activity of the states and are between zero and one such that the most active state has the highest occupancy rate. We set a threshold equal to the median of the occupancy rates. The states with occupancy rates greater than the threshold are maintained and are referred to as the active set of states.

For the states in the active set of states, we compute the state projections. State projections are computed by multiplying the state posterior probabilities (Supplementary Fig. 3b) to the daily cluster-grid mobile activity representations (Supplementary Fig. 3c). The state projections are referred to as the spatiotemporal patterns. The spatiotemporal patterns belonging to the active set of states are referred to as the major spatiotemporal patterns in data. Next, we compute the correlation between the (daily) major spatiotemporal patterns and the COVID-19 admission data on the daily number of admitted patients. Those spatiotemporal patterns that are statistically positively or negatively correlated with the daily number of admitted patients are maintained and are referred to as the correlated spatiotemporal patterns. Note, that we discard the spatiotemporal patterns that are not statistically correlated. The correlated spatiotemporal patterns are those latent patterns in mobile activity data that are most related to the COVID-19 admission data. The correlated spatiotemporal patterns are shown in Supplementary Fig. 4a and their correlation scores are shown in Supplementary Fig. 4b. Note that while here we used Pearson correlation which is a measure of linear correlation, alternatively non-linear measures of correlation could be used for this purpose.

Finally, we build a spatiotemporal memory from the extracted correlated spatiotemporal patterns by stacking the correlated spatiotemporal patterns into a matrix. The size of the memory matrix varies based on the number of correlated spatiotemporal patterns extracted from data.

### Predictive model

As shown in Fig. 1c, the predictive model takes as its inputs historical frames of the spatiotemporal memory matrix, historical data on the vaccination data, and COVID-19 admission data. It then produces forecasts for the duration of the forecast window. Here, we use a multilayer perceptron (MLP) regressor as our choice of predictive model. MLP belongs to the class of fully connected neural networks. The use of MLP as a simple neural network architecture in favor of recurrent neural nets (RNNs), which are designed for modelling temporal data, is motivated by the fact that the input to the MLP already contains carefully engineered short-to-long term spatiotemporal features. Being a non-temporal model, MLP would preserve the structure while RNNs will not. Table 1 shows the design choices in construction of the MLP neural network. The same neural network architecture was used for SU and SÄS. Note that, the dimensionality of the input layer depends on the size of the input spatiotemporal memory matrix which is determined automatically by the spatiotemporal model depending on the data under consideration for the given analysis date.

#### Reducing degeneracy problem of the forecast models

To reduce the degeneracy problem, we modified the loss function by adding a regularization term to the loss function. The regularization term discourages forecasts that are similar to the past and encourages uncovering novel trends.

Let *L* denote the loss between the true number of admitted patients $Y_t$ and the predicted number of admitted patients $\hat{Y}_t$ at the day *t*, defined as:

2 $$\begin{aligned} L = \sum _{t=1}^T \ell \left( Y_t, \hat{Y}_t \right) , \end{aligned}$$

where *T* is the number of days in the training set, and $\ell$ denotes the mean-square-error between true and predicted number of admitted patients. We obtain a regularized version of the loss defined as:

3 $$\begin{aligned} L_{\mathrm {regularized}} = \ell (Y_{0:W}, \hat{Y}_{0:W}) + \sum _{t=1}^T (1-\beta ) \ell \left( Y_{iW:(i+1)W}, \hat{Y}_{iW:(i+1)W} \right) - \beta \ell \left( \hat{Y}_{(i-1)W:iW}, \hat{Y}_{iW:(i+1)W} \right) . \end{aligned}$$

In above equation, *W* is the length of the forecast window, ${Y_{iW:(i+1)W}}$ denotes the *W* elements of the true number of admitted patients at the frame *i*, ${\hat{Y}_{iW:(i+1)W}}$ is the corresponding predicted number of admitted patients, and $\beta$ is a regularization term where ${0\le \beta <0.5}$. The second term in the summation is a regularization term which penalizes the loss if the forecasts at frame *i* is similar to the ones at the previous frame, $i-1$. The optimal value of $\beta$ shall be chosen thorough cross validation. In all analysis, $\beta$ was set to 0.25. The ML neural network is trained using the regularized loss in Eq. (3).

#### Inclusion of the antibody data in forecast model

The effect of antibody test data was included in the forecast model indirectly through the spatiotemporal model. For that, the user mobile activities are scaled up through an antibody curve computed from the cumulative statistics on the antibody positive rate, as

4 $$\begin{aligned} Z_t \leftarrow \left( 1 + \gamma _t\right) Z_t, \end{aligned}$$

where $Z_t$ denotes the average user activities at a given day *t*, and $\gamma _t$ is the cumulative number of antibody positive rate at the day *t*. The antibody curve is shown in Fig. 10a.

#### Inclusion of the vaccination data in forecast model

The effect of vaccination data was included in the forecast model through the predictive model, as shown in Fig. 9c. Before introduction of the vaccination data to the forecast model pipeline, the predictive model at the learning phase was trained to learn the following problem

5 $$\begin{aligned} \hat{Y}_t = f(X_t), \quad \forall t, \end{aligned}$$

where *f* is the predictive model which takes $X_t$ and produces the forecasts $\hat{Y}_t$. After introduction of the vaccination data, the predictive model was trained to solve instead the following problem,

6 $$\begin{aligned} \hat{Y}_t = (1-g(\alpha _t )) f(X_t), \quad \forall t \end{aligned}$$

where *g* is the vaccination efficacy model, and $\alpha _t$ is the vaccination rate at the day *t* computed from the cumulative number of vaccinated individuals normalized by the population. Effect of vaccination data was included in the forecast models from 2021-04-26. Up until 2021-05-25, we considered linear effect, that means ${g(\alpha _t) \approx \alpha _t}$. However, since 2021-05-25 onward, we considered a nonlinear effect where the vaccination efficacy model *g* was learned from the vaccination efficacy curve computed from vaccination campaign in Israel^24^. Figure 10b shows the vaccination efficacy curve from which vaccination efficacy model *g* was learned by fitting a polynomial model.

**Table 1:** MLP neural network used as the predictive model of the forecasts models.

Net	Number of units	Activation function	Batch normalization	Drop out
Input Layer	$D_{\mathrm {input}}$	ReLU	True	0.2
Hidden Layer 1	50	ReLU	True	0.2
Hidden Layer 2	50	ReLU	True	0.2
Hidden Layer 3	50	ReLU	True	0.2
Output Layer	1	Linear	False	False
Loss Function	Equation 3			
Optimizer	Adam^33^, learning rate = 0.001			

In using vaccination data in the models, we considered only the effect of dose one. This is explained as follows. Initially, we had only access to dose-one data. Once dose two data became available, we trained models both based on dose one and dose two. However, in our validation of the forecast models, data from dose one resulted in models with a tendency in slight over-estimation of the number of admitted patients while data from dose two resulted in models with a tendency in under-estimation of the number of admitted patients. This could have been due to our approach in incorporation of the vaccination data as an external factor into the models. While dose might be seen as a more reliable external factor, the fact that it was not available from the beginning created challenges in development and validation of the models (Fig. 11). We ultimately decided in using only dose one as there was in general a preference around over-estimation than under-estimation of the number of admitted patients.

## Supplementary Information

Supplementary Information.

## Data Availability

For both anonymous and aggregated mobile network data and vaccination data, we obtained permissions to use. For using anonymous and aggregated mobile network data, we have signed legal agreements with Telia Sverige AB. The vaccination data were provided directly from Swedish healthcare, Region Västra Götaland. Both Telia Sverige AB and Region Västra Götaland were part of the project. Vaccination data can be shared upon request. Anonymous and aggregated mobile network data are confidential and cannot be shared publicly. Anonymous and aggregated mobility data from Telia Sverige AB is commercially available as part of the service “Telia Crowd Insights.” For more information, please see https://business.teliacompany.com/crowd-insights.
