# Supplementary material for: Development of forecast models for COVID-19 hospital admissions using anonymized and aggregated mobile network data
Source: Sci Rep. 2022 Oct 22;12:17726. doi: 10.1038/s41598-022-22350-6 (PMC9588002; doi:10.1038/s41598-022-22350-6)
Supplement: Supplementary file 1 — Supplementary Information. [file 41598_2022_22350_MOESM1_ESM.pdf]

Development of Forecast Models for COVID-19 Hospital Admissions using Mobile Network Data: A Privacy-Preserving Approach (Supplementary Materials)

Jalil Taghia[1,*], Valentin Kulyk[1], Selim Ickin[1], Mats Folkesson[1], Cecilia Nyström[2], Kristofer Ågren[3], Thomas Brezicka[4], Tore Vingare[5], Julia Karlsson[5], Ingrid Fritzell[5], Ralph Harlid[6], Bo Palaszewski[7], Magnus Kjellberg[8], and Jörgen Gustafsson[1,*]

[1]Ericsson Research, Ericsson, Kista, 164 40, Sweden
[2]Ericsson Business Area Managed Services, Ericsson, Kista, 164 40, Sweden
[3]Telia Company AB, 169 94 Solna, Sweden
[4]Sahlgrenska University Hospital, Department of Quality and Patient Safety, Gothenburg, 413 45, Sweden
[5]Sahlgrenska University Hospital, Department of Analysis and Project Management, Gothenburg 413 45, Sweden
[6]Södra Älvsborgs Sjukhus, Sjukhusledningen, Borås, 501 82, Sweden
[7]Department of Data Management and Analysis, Västra Götalandsregionen, Gothenburg, 405 44, Sweden
[8]Sahlgrenska University Hospital, AI Competence Center, Gothenburg, 413 45, Sweden
[*]corresponding author(s): Jalil Taghia and Jörgen Gustafsson (jalil.taghia@ericsson.com, jorgen.gustafsson@ericsson.com)

References

1. OpenStreetMap contributors. Planet dump retrieved from https://planet.osm.org . https://www.openstreetmap.org (2017).

2. Taghia, J. *et al.* Uncovering hidden brain state dynamics that regulate performance and decision-making during cognition. *Nat. Commun.* **9** (2018).

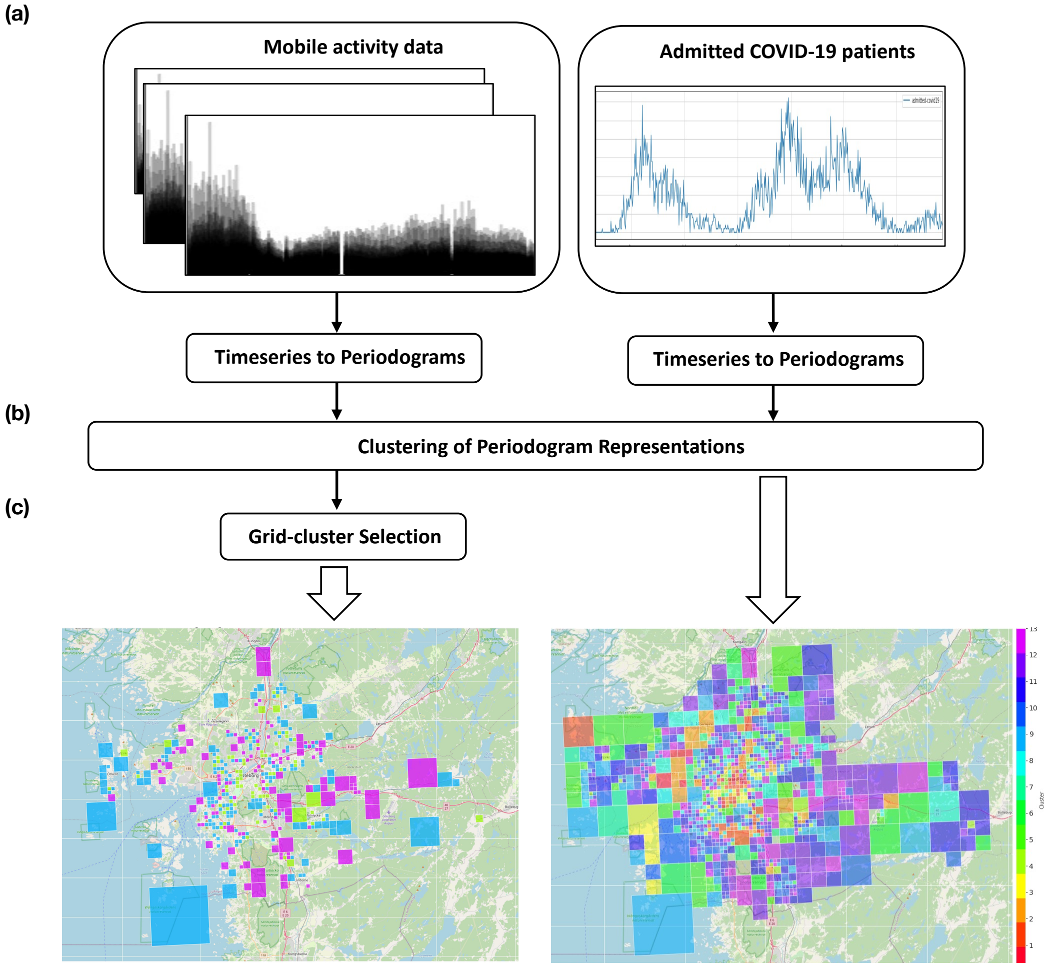

Supplementary Figure 1. Steps taken by grid selection model. (a) Generation of the periodogram representations from mobile activity and COVID-19 timeseries data. (b) Clustering of the periodogram representations into a number of clusters where the optimal number of clusters are decided based on the information theoretic measure of interaction information. (c) Grid-cluster selection identifies the clusters of grids that are most relevant to the COVID-19 data by choosing the clusters with the highest negative interaction information scores.

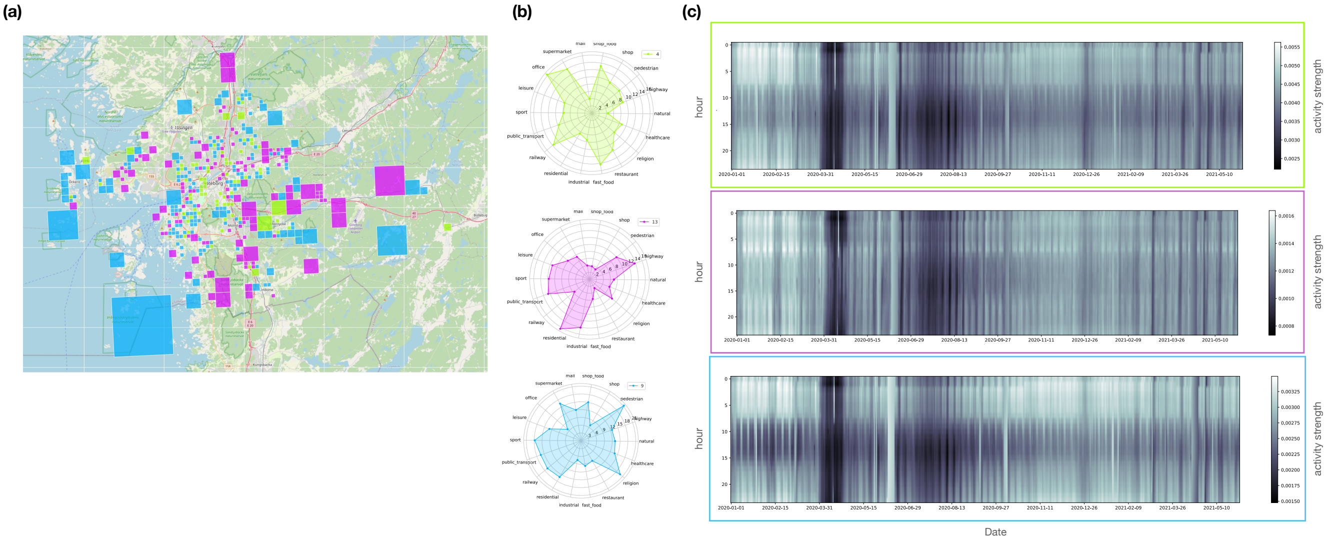

Supplementary Figure 2. (a) The grid clusters provided by the grid selection model. (b) Cluster description using tags taken from OpenStreetMap[1]. (c) The hourly cluster-grid mobile activity representations. Examples are for Sahlgrenska University hospital using historical data for the analysis date on 2021-06-07.

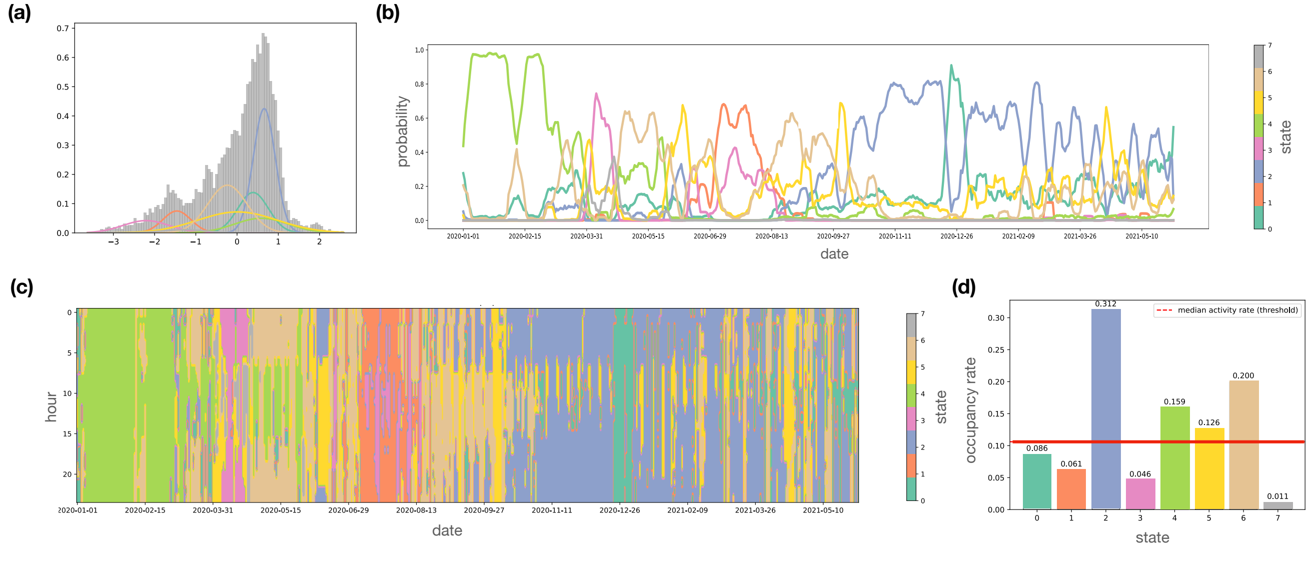

Supplementary Figure 3. Measures extracted from temporal modeling of mobile activity data using Bayesian switching dynamical systems (BSDS)[2]. (a) State distributions where each state is presented by a Gaussian distribution. (b) The state posterior probabilities. (c) The temporal evolution of states indicating state activities at a given time and at a given day. (d) Occupancy rate of each state, computed from temporal evolution of states. The occupancy rate of states indicates the activity of the states. The solid red line indicates the threshold which is equal to the median of the occupancy rates. The states with occupancy rates greater than the threshold are referred to as the active set of states. Examples are for Sahlgrenska University hospital using historical data for the analysis date on 2021-06-07.

(a)

(b)

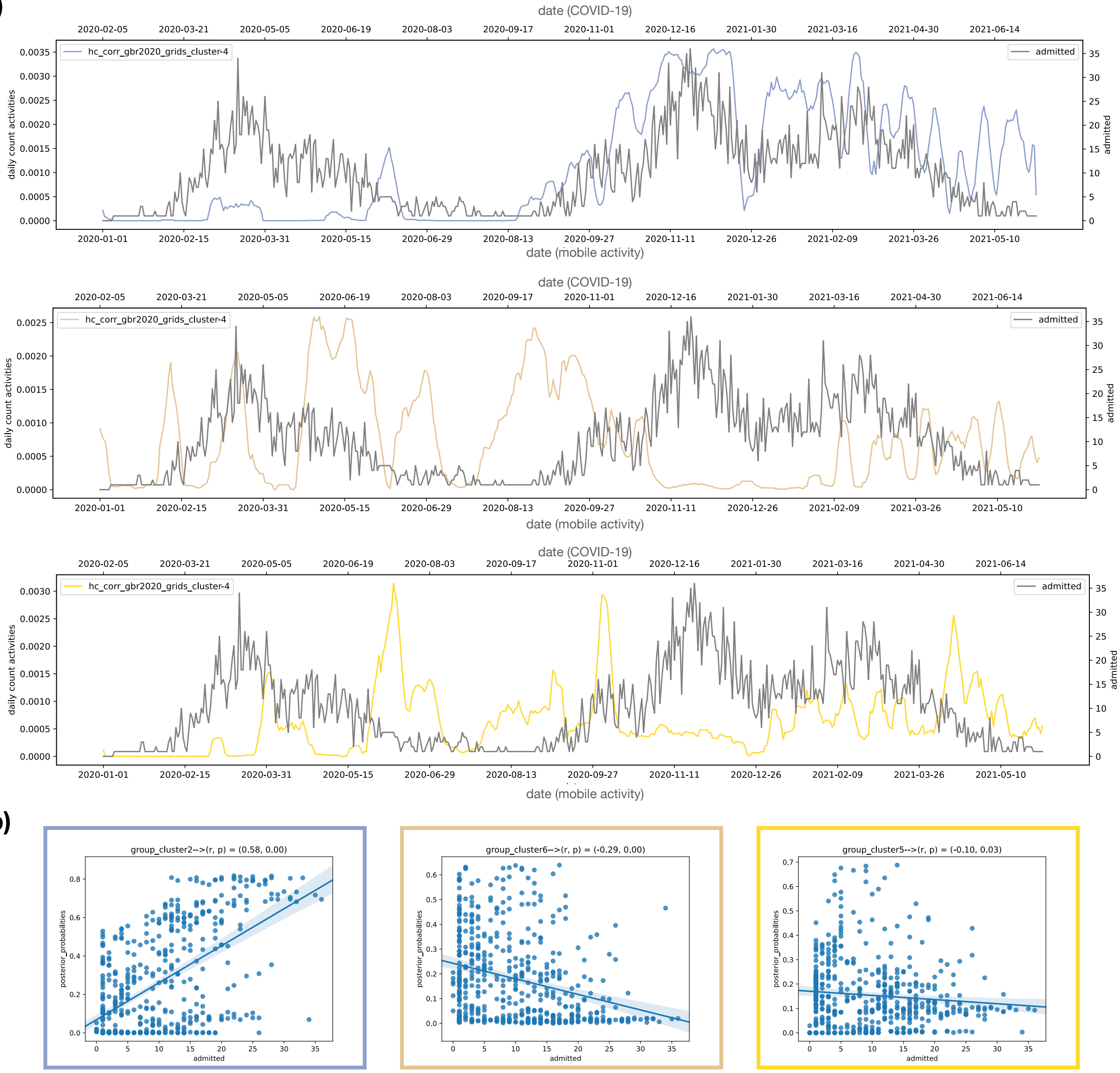

Supplementary Figure 4. (a) The correlated spatiotemporal components from the active set of states, Supplementary Figure 3.d. The grey line shows the number of admitted COVID-19 admitted patients. There is a 35-day lag between the mobile activity data and COVID-19 data. (b) Correlation scores between the correlated spatiotemporal components and the daily number of admitted COVID-19 patients. Examples are for Sahlgrenska University hospital using historical data for the analysis date on 2021-06-07.
